# Author Correction: Vitamin D status and severity of COVID-19

**DOI:** 10.1038/s41598-023-28993-3

**Published:** 2023-01-31

**Authors:** Nete Munk Nielsen, Thor Grønborg Junker, Sanne Grundvad Boelt, Arieh S. Cohen, Kassandra L. Munger, Egon Stenager, Alberto Ascherio, Lasse Boding, Anders Hviid

**Affiliations:** 1 Department of Epidemiology Research, Statens Serum Institut, Copenhagen, Denmark; 2 Focused Research Unit in Neurology, Department of Neurology, Hospital of Southern Jutland, University of Southern Denmark, Aabenraa, Denmark; 3 Section for Clinical Mass Spectrometry, Danish Center for Neonatal Screening, Department of Congenital Disorders, Statens Serum Institut, Copenhagen, Denmark; 4 iPSYCH, The Lundbeck Foundation Initiative for Integrative Psychiatric Research, Copenhagen, Denmark; 5 Test Center Denmark, Statens Serum Institut, Copenhagen, Denmark; 6 Department of Nutrition, Harvard T.H. Chan School of Public Health, Boston, MA USA; 7 Multiple Sclerosis Clinic of Southern Jutland (Sønderborg, Kolding, Esbjerg), Department of Neurology, Hospital of Southern Jutland, University of Southern Denmark, Sønderborg, Denmark; 8 Department of Epidemiology, Harvard T.H. Chan School of Public Health, Boston, MA USA; 9 Channing Division of Network Medicine, Brigham and Women’s Hospital and Harvard Medical School, Boston, MA USA; 10 The Danish National Biobank, Statens Serum Institut, Copenhagen, Denmark; 11 Pharmacovigilance Research Centre, Department of Drug Design and Pharmacology, University of Copenhagen, Copenhagen, Denmark

Correction to: *Scientific Reports* 10.1038/s41598-022-21513-9, published online 17 November 2022

Sanne Grundvald Boelt was omitted from the author list in the original version of this Article.

The Author Contributions section now reads:

“A.A. contributed to the conception and idea of the work. N.M.N., A.S.C., L.B. and A.A. contributed to the design of the study. N.M.N., A.S.C., L.B. S.G.B contributed to the acquisition of data. N.M.N., A.H., L.B., S.G.B and T.G.J. contributed to the analyses of the data. All authors contributed to the interpretation of the data and the results in the manuscript. N.M.N. drafted the manuscript, made Fig. 1, T.G.J. made Fig. 2. All the authors revised the manuscript critically and has approved the final version.”

The original Article has been corrected.

